# Durability of Original Monovalent mRNA Vaccine Effectiveness Against COVID-19 Omicron–Associated Hospitalization in Children and Adolescents — United States, 2021–2023

**DOI:** 10.15585/mmwr.mm7315a2

**Published:** 2024-04-18

**Authors:** Laura D. Zambrano, Margaret M. Newhams, Regina M. Simeone, Amanda B. Payne, Michael Wu, Amber O. Orzel-Lockwood, Natasha B. Halasa, Jemima M. Calixte, Pia S. Pannaraj, Kanokporn Mongkolrattanothai, Julie A. Boom, Leila C. Sahni, Satoshi Kamidani, Kathleen Chiotos, Melissa A. Cameron, Aline B. Maddux, Katherine Irby, Jennifer E. Schuster, Elizabeth H. Mack, Austin Biggs, Bria M. Coates, Kelly N. Michelson, Katherine E. Bline, Ryan A. Nofziger, Hillary Crandall, Charlotte V. Hobbs, Shira J. Gertz, Sabrina M. Heidemann, Tamara T. Bradford, Tracie C. Walker, Stephanie P. Schwartz, Mary Allen Staat, Samina S. Bhumbra, Janet R. Hume, Michele Kong, Melissa S. Stockwell, Thomas J. Connors, Melissa L. Cullimore, Heidi R. Flori, Emily R. Levy, Natalie Z. Cvijanovich, Matt S. Zinter, Mia Maamari, Cindy Bowens, Danielle M. Zerr, Judith A. Guzman-Cottrill, Ivan Gonzalez, Angela P. Campbell, Adrienne G. Randolph, Overcoming COVID-19 Investigators, Overcoming COVID-19 Investigators, Meghan Murdock, Heather Kelley, Candice Colston, Ronald C. Sanders, Laura Miron, Masson Yates, Ashlyn Madding, Alexa Dixon, Michael Henne, Kathleen Sun, Jazmin Baez Maidana, Natalie Triester, Jaycee Jumarang, Daniel Hakimi, Kennis-Grace Mrotek, Liria Muriscot Niell, Natasha Baig, Elizabeth Temte, Lexi Petruccelli, Heidi Sauceda, Nicolette Gomez, Mark D. Gonzalez, Caroline R. Ciric, Jong-Ha C. Choi, Elizabeth G. Taylor, Grace X. Li, Nadine Baida, Heather E. Price, Mary Stumpf, Suden Kucukak, Eve Listerud, Maya Clark, Rylie Dittrich, Allison Zaff, Patrick Moran, Jessica C. Peterson, Noelle M. Drapeau, Lora Martin, Lacy Malloch, Maygan Martin, Cameron Sanders, Kayla Patterson, Melissa Sullivan, Shannon Pruitt, Elizabeth Ricciardi, Celibell Y. Vargas, Raul A. Silverio Francisco, Ana Valdez de Romero, Sheila Joshi, Merry Tomcany, Nicole Twinem, Chelsea C. Rohlfs, Amber Wolfe, Rebecca Douglas, Kathlyn Phengchomphet, Jenny Bush, Alanah Mckelvey, Mickael Boustany, Fatima A. Mohammed, Laura S. Stewart, Kailee Fernandez, Leenah Abojaib, Molly J. Kyles, Amanda Adler

**Affiliations:** ^1^Coronavirus and Other Respiratory Viruses Division, National Center for Immunization and Respiratory Diseases, CDC; ^2^Department of Anesthesiology, Critical Care, and Pain Medicine, Boston Children’s Hospital, Boston, Massachusetts; ^3^Division of Pediatric Infectious Diseases, Department of Pediatrics, Vanderbilt University Medical Center, Nashville, Tennessee; ^4^Division of Infectious Diseases, Children’s Hospital Los Angeles, Los Angeles, California; ^5^Department of Pediatrics, University of California, San Diego, San Diego, California; ^6^Division of Pediatric Infectious Diseases, Department of Pediatrics, Children’s Hospital Los Angeles, Los Angeles, California; ^7^Department of Pediatrics, Baylor College of Medicine, Immunization Project, Texas Children’s Hospital, Houston, Texas; ^8^The Center for Childhood Infections and Vaccines of Children’s Healthcare of Atlanta, Atlanta, Georgia; ^9^Department of Pediatrics, Emory University School of Medicine, Atlanta, Georgia; ^10^Division of Critical Care Medicine, Department of Anesthesiology and Critical Care, Children’s Hospital of Philadelphia, Philadelphia, Pennsylvania; ^11^Division of Pediatric Hospital Medicine, UC San Diego-Rady Children’s Hospital, San Diego, California; ^12^Department of Pediatrics, Section of Critical Care Medicine, University of Colorado School of Medicine, Aurora, Colorado; ^13^Children’s Hospital Colorado, Aurora, Colorado; ^14^Section of Pediatric Critical Care, Department of Pediatrics, Arkansas Children's Hospital, Little Rock, Arkansas; ^15^Division of Pediatric Infectious Diseases, Department of Pediatrics, Children’s Mercy Kansas City, Kansas City, Missouri; ^16^Division of Pediatric Critical Care Medicine, Medical University of South Carolina, Charleston, South Carolina; ^17^Department of Pediatrics, Northwestern University Feinberg School of Medicine, Chicago, Illinois; ^18^Division of Critical Care Medicine, Ann & Robert H. Lurie Children’s Hospital of Chicago, Chicago, Illinois; ^19^Division of Pediatric Critical Care Medicine, Nationwide Children’s Hospital, Columbus, Ohio; ^20^Division of Critical Care Medicine, Department of Pediatrics, Akron Children’s Hospital, Akron, Ohio; ^21^Division of Pediatric Critical Care, Department of Pediatrics, University of Utah, Salt Lake City, Utah; ^22^Primary Children’s Hospital, Salt Lake City, Utah; ^23^Department of Pediatrics, Division of Infectious Diseases, University of Mississippi Medical Center, Jackson, Mississippi; ^24^Division of Pediatric Critical Care, Department of Pediatrics, Cooperman Barnabas Medical Center, Livingston, New Jersey; ^25^Division of Pediatric Critical Care Medicine, Children’s Hospital of Michigan, Central Michigan University, Detroit, Michigan; ^26^Department of Pediatrics, Division of Cardiology, Louisiana State University Health Sciences Center, New Orleans, Louisiana; ^27^Children’s Hospital of New Orleans, New Orleans, Louisiana; ^28^Department of Pediatrics, University of North Carolina at Chapel Hill Children's Hospital, Chapel Hill, North Carolina; ^29^Department of Pediatrics, University of Cincinnati College of Medicine, Cincinnati Children's Hospital Medical Center, Cincinnati, Ohio; ^30^Ryan White Center for Pediatric Infectious Disease and Global Health, Department of Pediatrics, Indiana University School of Medicine, Indianapolis, Indiana; ^31^Division of Pediatric Critical Care, University of Minnesota Masonic Children’s Hospital, Minneapolis, Minnesota; ^32^Division of Pediatric Critical Care Medicine, Department of Pediatrics, University of Alabama at Birmingham, Birmingham, Alabama; ^33^Division of Child and Adolescent Health, Department of Pediatrics, Vagelos College of Physicians and Surgeons, New York, New York; ^34^Department of Population and Family Health, Mailman School of Public Health Columbia University, New York, New York; ^35^New York-Presbyterian Morgan Stanley Children’s Hospital; New York, New York; ^36^Division of Critical Care and Hospital Medicine, Department of Pediatrics, Vagelos College of Physicians and Surgeons, Columbia University, New York, New York; ^37^Division of Pediatric Critical Care, Department of Pediatrics, Children's Nebraska, Omaha, Nebraska; ^38^Division of Pediatric Critical Care Medicine, Department of Pediatrics, C.S. Mott Children’s Hospital, Ann Arbor, Michigan; ^39^Divisions of Pediatric Infectious Diseases and Pediatric Critical Care Medicine, Department of Pediatric and Adolescent Medicine, Mayo Clinic, Rochester, Minnesota; ^40^Division of Critical Care Medicine, UCSF Benioff Children's Hospital, Oakland, California; ^41^Department of Pediatrics, Divisions of Critical Care Medicine and Allergy, Immunology, and Bone Marrow Transplant, University of California San Francisco, San Francisco, California; ^42^Department of Pediatrics, Division of Critical Care Medicine, University of Texas Southwestern, Children's Medical Center Dallas, Texas; ^43^Division of Pediatric Infectious Diseases, Department of Pediatrics, Seattle Children's Hospital, Seattle, Washington; ^44^Department of Pediatrics, Division of Infectious Diseases, Oregon Health & Science University, Portland, Oregon; ^45^Division of Pediatric Infectious Diseases, Department of Pediatrics, University of Miami Miller School of Medicine, Miami, Florida; ^46^Department of Anaesthesia, Harvard Medical School, Boston, Massachusetts; ^47^Department of Pediatrics, Harvard Medical School, Boston, Massachusetts.; Children’s of Alabama, Birmingham, Alabama; Children’s of Alabama, Birmingham, Alabama; Children’s of Alabama, Birmingham, Alabama; Arkansas Children’s Hospital, Little Rock, Arkansas; Arkansas Children’s Hospital, Little Rock, Arkansas; Arkansas Children’s Hospital, Little Rock, Arkansas; Arkansas Children’s Hospital, Little Rock, Arkansas; Arkansas Children’s Hospital, Little Rock, Arkansas; Rady Children’s Hospital, San Diego, California; UCSF Benioff Children’s Hospital, San Francisco, California; UCSF Benioff Children’s Hospital, San Francisco, California; UCSF Benioff Children’s Hospital Oakland, Oakland, California; Children’s Hospital Los Angeles, Los Angeles, California; Children’s Hospital Los Angeles, Los Angeles, California; Children’s Hospital Los Angeles, Los Angeles, California; Children’s Hospital Los Angeles, Los Angeles, California; Children’s Hospital Colorado, Aurora, Colorado; Children’s Hospital Colorado, Aurora, Colorado; Children’s Hospital Colorado, Aurora, Colorado; Children’s Hospital Colorado, Aurora, Colorado; Holtz Children’s Hospital, Miami, Florida; Emory University School of Medicine and Children's Healthcare of Atlanta, Atlanta, Georgia; Emory University School of Medicine and Children's Healthcare of Atlanta, Atlanta, Georgia; Emory University School of Medicine and Children's Healthcare of Atlanta, Atlanta, Georgia; Emory University School of Medicine and Children's Healthcare of Atlanta, Atlanta, Georgia; Emory University School of Medicine and Children's Healthcare of Atlanta, Atlanta, Georgia; Emory University School of Medicine and Children's Healthcare of Atlanta, Atlanta, Georgia; Ann & Robert H. Lurie Children’s Hospital of Chicago, Chicago, Illinois; Riley Hospital for Children, Indianapolis, Indiana; Boston Children’s Hospital, Boston, Massachusetts; Boston Children’s Hospital, Boston, Massachusetts; Boston Children’s Hospital, Boston, Massachusetts; Boston Children’s Hospital, Boston, Massachusetts; Boston Children’s Hospital, Boston, Massachusetts; University of Michigan C.S. Mott Children’s Hospital, Ann Arbor, Michigan; Mayo Clinic, Rochester, Minnesota; Mayo Clinic, Rochester, Minnesota; Children’s Hospital of Mississippi, Jackson, Mississippi; Children’s Hospital of Mississippi, Jackson, Mississippi; Children’s Hospital of Mississippi, Jackson, Mississippi; Children’s Hospital of Mississippi, Jackson, Mississippi; Children’s Hospital of Mississippi, Jackson, Mississippi; Children’s Mercy Kansas City, Kansas City, Missouri; Children’s Mercy Kansas City, Kansas City, Missouri; Cooperman Barnabas Medical Center, Livingston, New Jersey; Columbia University Irving Medical Center, New York, New York; Columbia University Irving Medical Center, New York, New York; Columbia University Irving Medical Center, New York, New York; University of North Carolina at Chapel Hill, Chapel Hill, North Carolina; Akron Children’s Hospital, Akron, Ohio; Akron Children’s Hospital, Akron, Ohio; Cincinnati Children’s Hospital, Cincinnati, Ohio; Nationwide Children’s Hospital, Columbus, Ohio; Children’s Hospital of Philadelphia, Philadelphia, Pennsylvania; Children’s Hospital of Philadelphia, Philadelphia, Pennsylvania; Children’s Hospital of Philadelphia, Philadelphia, Pennsylvania; Children’s Hospital of Philadelphia, Philadelphia, Pennsylvania; Children’s Hospital of Philadelphia, Philadelphia, Pennsylvania; MUSC Children’s Health, Charleston, South Carolina; Monroe Carell Jr. Children’s Hospital at Vanderbilt, Nashville, Tennessee; Monroe Carell Jr. Children’s Hospital at Vanderbilt, Nashville, Tennessee; Texas Children’s Hospital and Baylor College of Medicine, Houston, Texas; Texas Children’s Hospital and Baylor College of Medicine, Houston, Texas; Seattle Children’s Hospital, Seattle, Washington

**Summary**

**What is already known about this topic?**

COVID-19 vaccination was shown to be effective against pediatric COVID-19 hospitalization before the emergence of the Omicron variant.

**What is added by this report?**

During December 19, 2021–October 29, 2023, receipt of ≥2 doses of an original monovalent mRNA COVID-19 vaccine was 52% effective against pediatric COVID-19 hospitalization and 57% effective against critical illness related to COVID-19, when the last dose was received within the 4 months preceding hospitalization, but protection decreased over time.

**What are the implications for public health practice?**

These findings support existing recommendations that children and adolescents aged 5–18 years remain up to date with COVID-19 vaccination given low vaccination coverage and waning effectiveness over time against COVID-19–related hospitalizations.

## Abstract

Pediatric COVID-19 vaccination is effective in preventing COVID-19–related hospitalization, but duration of protection of the original monovalent vaccine during SARS-CoV-2 Omicron predominance merits evaluation, particularly given low coverage with updated COVID-19 vaccines. During December 19, 2021–October 29, 2023, the Overcoming COVID-19 Network evaluated vaccine effectiveness (VE) of ≥2 original monovalent COVID-19 mRNA vaccine doses against COVID-19–related hospitalization and critical illness among U.S. children and adolescents aged 5–18 years, using a case-control design. Too few children and adolescents received bivalent or updated monovalent vaccines to separately evaluate their effectiveness. Most case-patients (persons with a positive SARS-CoV-2 test result) were unvaccinated, despite the high frequency of reported underlying conditions associated with severe COVID-19. VE of the original monovalent vaccine against COVID-19–related hospitalizations was 52% (95% CI = 33%–66%) when the most recent dose was administered <120 days before hospitalization and 19% (95% CI = 2%–32%) if the interval was 120–364 days. VE of the original monovalent vaccine against COVID-19–related hospitalization was 31% (95% CI = 18%–43%) if the last dose was received any time within the previous year. VE against critical COVID-19–related illness, defined as receipt of noninvasive or invasive mechanical ventilation, vasoactive infusions, extracorporeal membrane oxygenation, and illness resulting in death, was 57% (95% CI = 21%–76%) when the most recent dose was received <120 days before hospitalization, 25% (95% CI = –9% to 49%) if it was received 120–364 days before hospitalization, and 38% (95% CI = 15%–55%) if the last dose was received any time within the previous year. VE was similar after excluding children and adolescents with documented immunocompromising conditions. Because of the low frequency of children who received updated COVID-19 vaccines and waning effectiveness of original monovalent doses, these data support CDC recommendations that all children and adolescents receive updated COVID-19 vaccines to protect against severe COVID-19.

## Introduction

mRNA COVID-19 vaccines have been recommended for U.S. children and adolescents aged ≥5 years since November 2021^†^ (*1*). Two doses of Pfizer-BioNTech (BNT162b2) vaccine protected against COVID-19–related hospitalizations before and after emergence of the SARS-CoV-2 Delta variant (*2*,*3*). Throughout Omicron variant predominance (beginning in December 2021), estimated pediatric COVID-19 vaccine effectiveness (VE) of the original monovalent vaccine was lower (*2*,*4*). This analysis evaluated durability of effectiveness of original monovalent vaccines, which were only available before September 2022, against COVID-19–related hospitalization among children and adolescents aged 5–18 years during December 19, 2021–October 29, 2023, when the SARS-CoV-2 Omicron variant predominated.

## Methods

### Study Participants

VE of ≥2 original monovalent COVID-19 vaccine doses^§^ against COVID-19–related hospitalizations (December 19, 2021–October 29, 2023^¶^) across 34 Overcoming COVID-19 Network sites** was evaluated using a case-control design according to previously described methods (*2*,*3*). Case-patients were children and adolescents aged 5–18 years who were hospitalized for acute COVID-19 and received a positive SARS-CoV-2 test result.^††^ Control patients hospitalized for COVID-19–like illness were matched to case-patients by site, age group, and admission date, but received a negative SARS-CoV-2 test result.^§§^ Critical COVID-19–related illness was defined as receipt of noninvasive or invasive mechanical ventilation, vasoactive infusions, extracorporeal membrane oxygenation, and illness resulting in death. Children and adolescents were a priori excluded from the analysis if they 1) received their most recent dose ≥365 days before hospitalization, 2) had an incomplete COVID-19 mRNA primary vaccination series, 3) had a COVID-19 hospitalization within the preceding 60 days, 4) had an unverifiable vaccination status, or 5) received a positive influenza test result.^¶¶^ Given subsequent findings of low (3%) bivalent vaccination coverage and no reported receipt of updated (2023–2024 formula) monovalent doses, children who received updated formulations were post hoc excluded from VE analyses. 

### Statistical Analysis and Vaccine Effectiveness Estimation 

Bivariate associations between sociodemographic factors and both case or control status and vaccination status among case- and control patients were assessed using chi-square tests for binomial or categorical variables or Wilcoxon rank-sum tests for continuous variables. VE was estimated among all hospitalized patients and among patients without documented immunocompromising conditions*** and calculated as (1 − adjusted odds ratio) × 100% by time between last vaccine dose and hospitalization and by age,^†††^ using multivariable logistic regression,^§§§^ including hospital site as a repeated effect using generalized estimating equations, and adjusting for the presence of one or more underlying medical condition, age (in years), month and year of hospitalization, U.S. Census Bureau region of hospital, social vulnerability index (SVI; i.e., continuous ranging from 0–1, with higher scores indicating increased vulnerability), and race and ethnicity. SAS software (version 9.4; SAS Institute) was used to conduct all analyses. This activity was reviewed by CDC, deemed not research, and conducted consistent with applicable federal law and CDC policy.^¶¶¶^

## Results

### Characteristics of Enrolled Population

During December 19, 2021–October 29, 2023, a total of 3,348 patients were enrolled, including 1,551 (46%) case-patients and 1,797 (54%) control patients.**** Only 3% of case-patients and of control patients had received bivalent COVID-19 vaccine, and none reported receipt of an updated monovalent dose; therefore, VE for these specific formulations could not be estimated. Case- and control patients were similar in age, sex, hospital U.S. Census Bureau region,^††††^ presence of any underlying respiratory condition (e.g., asthma or chronic lung disease), and clinical support received (Table 1). The presence of at least one underlying health condition was more common among case-patients (82%) than among control patients (73%) (p-value <0.001). Critical illness occurred in 294 (19%) case-patients and 322 (18%) control patients (p = 0.43). Patients living in lower SVI areas were more frequently vaccinated (Table 2).

**TABLE 1:** Characteristics of children and adolescents aged 5–18 years hospitalized with a COVID-19–like illness and a positive SARS-CoV-2 test result (case-patients) or a negative SARS-CoV-2 test result (control patients) — Overcoming COVID-19 Network, 34 pediatric hospitals, 26 states, December 19, 2021–October 29, 2023

Characteristic (no. with known information)	No. (%)		p-value*
	Case-patients (n = 1,551)	Control patients (n = 1,797)	
**Age group, yrs**			
5–11	853 (55)	1,042 (58)	0.08
12–18	698 (45)	755 (42)	
**Median age, yrs, IQR**	11.3 (7.7–15.1)	10.5 (7.3–14.7)	0.01
**Female sex**	712 (46)	857 (48)	0.59
**Race and ethnicity**			
Asian, non-Hispanic	71 (5)	41 (2)	<0.001
Black or African American, non-Hispanic	403 (26)	438 (24)	
White, non-Hispanic	571 (37)	675 (38)	
Hispanic or Latino, any race	406 (26)	485 (27)	
Multiple or other races, non-Hispanic	47 (3)	65 (4)	
Unknown	53 (3)	93 (5)	
**Median social vulnerability index, IQR (3,345)^†^**	0.58 (0.37–0.78)	0.57 (0.33–0.77)	0.10
**U.S. Census Bureau region** ^§^			
Northeast	253 (16)	272 (15)	0.35
Midwest	364 (23)	466 (26)	
South	565 (36)	628 (35)	
West	369 (24)	431 (24)	
**Circulating Omicron subvariant during hospitalization** ^¶^			
Omicron BA.1/BA.1.1	638 (41)	776 (43)	0.23
Omicron BA.2/BA.4/BA.5/XBB.1.5/XBB.1.6	913 (59)	1021 (57)	
**Underlying health conditions**			
None	275 (18)	489 (27)	<0.001
One or more	1,276 (82)	1,308 (73)	
Respiratory, including asthma	619 (40)	744 (41)	0.38
Cardiac	235 (15)	172 (10)	<0.001
Neurologic or neuromuscular	524 (34)	352 (20)	<0.001
Immunocompromising conditions**	273 (18)	165 (9)	<0.001
Endocrine, including diabetes	195 (13)	181 (10)	0.02
Multiple	526 (34)	382 (21)	<0.001
**COVID-19 vaccination status**			
Unvaccinated	1,137 (73)	1,210 (67)	<0.001
Original monovalent dose, 7–119 days before hospitalization**^††^**	94 (6)	207 (12)	
Original monovalent dose, 120−364 days before hospitalization	277 (18)	322 (18)	
Bivalent dose^§§^	43 (3)	58 (3)	
**Clinical course **			
ICU admission (3,347)	404 (26)	500 (28)	0.25
Critical illness (3,343)^¶¶^	294 (19)	322 (18)	0.43
Invasive mechanical ventilation	107 (7)	129 (7)	0.75
Noninvasive mechanical ventilation (BiPAP or CPAP) (3,347)	222 (14)	228 (13)	0.17
Vasoactive infusion	83 (5)	86 (5)	0.46
Extracorporeal membrane oxygenation	9 (1)	10 (1)	0.93
Died (3,343)	10 (1)	9 (1)	0.58
**Median hospital days (IQR) (3,340)**	4 (2–7)	4 (3–7)	0.79

**Abbreviations:** BiPAP = bilevel positive airway pressure; CPAP = continuous positive airway pressure; ICU = intensive care unit.

* Binomial or categorical variables were compared using chi-square tests of independence, and continuous variables were compared using Wilcoxon rank-sum tests.

^†^ The social vulnerability index is a scale (range = 0–1), reflecting a composite score of socioeconomic status, household characteristics, racial and ethnic minority status, and housing type and transportation. A lower score indicates lower social vulnerability, whereas a higher score indicates higher social vulnerability, which might predispose a population to worse health outcomes. https://www.atsdr.cdc.gov/placeandhealth/svi

^§^ https://www2.census.gov/geo/pdfs/maps-data/maps/reference/us_regdiv.pdf

^¶^ Periods of Omicron subvariant circulation were defined as follows: BA.1: December 19, 2021–March 19, 2022 and BA.2/BA.4/BA.5/XBB.1.5/XBB.1.6: March 20–October 29, 2022.

** Immunocompromising conditions included active or previous oncologic disorder or nononcologic immunosuppressive disorder (including solid organ transplant, HIV or AIDS, primary immunodeficiency, bone marrow transplant for nononcologic disease, and other disorder requiring treatment that suppresses the immune system).

^††^ All monovalent doses were original monovalent doses directed against wild type SARS-CoV-2. No child or adolescent had received an updated (2023–2024 formula) monovalent dose, authorized on September 11, 2023, before their hospitalization.

^§§^ Children and adolescents who received a bivalent dose were excluded from the primary vaccine effectiveness analysis because bivalent vaccination coverage was insufficient to calculate vaccine effectiveness for this formulation.

^¶¶^ Critical illness was defined as illness resulting in noninvasive ventilation, invasive mechanical ventilation, receipt of vasoactive infusions, extracorporeal membrane oxygenation, or death.

**TABLE 2:** Characteristics of COVID-19 case-patients and control patients with COVID-19–like illness, by vaccination status (N = 3,247) — Overcoming COVID-19 Network, 34 pediatric hospitals, 26 states, December 19, 2021–October 29, 2023

Characteristic (no. with known information if less than total N)	COVID-19 vaccination status, no. (%)					
	Case-patients (n = 1,508)*			Control patients (n = 1,739)^†^		
	Unvaccinated (n = 1,137)	Original monovalent dose, 7–364 d^§ ^(n = 371)	p-value^¶^	Unvaccinated (n = 1,210)	Original monovalent dose, 7–364 d^§ ^(n = 529)	p-value^¶^
**Median age, yrs (IQR)**	10.1 (7.2–14.0)	14.4 (11.0–16.6)	<0.001	9.3 (6.9–13.7)	13.3 (9.0–15.9)	<0.001
**Age group, yrs**						
5–11	718 (86)	114 (14)	<0.001	806 (80)	207 (20)	<0.001
12–18	419 (62)	257 (38)		404 (56)	322 (44)	
Sex (3,246)**						
Female	508 (74)	183 (26)	0.26	569 (68)	263 (32)	0.30
Male	628 (77)	188 (23)		641 (71)	266 (29)	
**Race and ethnicity**						
Asian, non-Hispanic	41 (59)	28 (41)	0.008	16 (40)	24 (60)	<0.001
Black or African American, non-Hispanic	312 (79)	83 (21)		327 (77)	97 (23)	
White, non-Hispanic	409 (73)	149 (27)		447 (69)	206 (32)	
Hispanic or Latino, any race	301 (77)	89 (23)		310 (66)	163 (34)	
Multiple or other races, non-Hispanic	37 (82)	8 (18)		50 (81)	12 (19)	
Unknown	37 (73)	14 (27)		60 (69)	27 (31)	
**Median SVI (IQR) (3,244)^††^**	0.60 (0.38–0.79)	0.55 (0.32–0.76)	0.01	0.59 (0.38–0.78)	0.52 (0.26–0.76)	<0.001
**U.S. Census Bureau region** ^§§^						
Northeast	154 (63)	91 (37)	<0.001	143 (55)	118 (45)	<0.001
Midwest	289 (82)	65 (18)		330 (73)	121 (27)	
South	457 (82)	99 (18)		476 (78)	135 (22)	
West	237 (67)	116 (33)		261 (63)	155 (37)	
**Underlying health conditions^¶¶^**						
None	214 (78)	59 (22)	0.20	342 (71)	138 (29)	0.35
One or more underlying condition	923 (75)	312 (25)		868 (69)	391 (31)	
Respiratory, including asthma	445 (75)	147 (25)	0.87	505 (71)	207 (29)	0.31
Cardiac	156 (69)	71 (31)	0.01	103 (62)	63 (38)	0.03
Neurologic or neuromuscular	379 (75)	127 (25)	0.75	207 (63)	122 (37)	0.004
Immunocompromising conditions***	188 (71)	76 (29)	0.08	99 (62)	61 (38)	0.03
Endocrine, including diabetes	131 (71)	54 (29)	0.12	115 (66)	59 (34)	0.29
Obesity	140 (75)	47 (25)	0.86	114 (67)	56 (33)	0.45
Multiple	355 (71)	145 (29)	0.005	231 (64)	130 (36)	0.01

**Abbreviations**: d = days before hospitalization; SVI = social vulnerability index.

* This analysis excludes 43 of 1,551 case-patients who received a bivalent vaccine dose.

^†^ This analysis excludes 58 of 1,797 control patients who received a bivalent vaccine dose.

^§^ All monovalent doses received before hospitalization were original monovalent vaccine doses directed against the original SARS-CoV-2 strain. No child or adolescent had received an updated (2023–2024 formula) monovalent vaccine dose, authorized on September 11, 2022, before hospitalization.

^¶^ Binomial or categorical variables were compared using chi-square tests of independence and continuous variables were compared using Wilcoxon rank-sum tests.

** One unvaccinated case-patient had sex noted as “other” and was excluded from this comparison.

^††^ SVI is a scale (range = 0–1), reflecting a composite score of socioeconomic status, household characteristics, racial and ethnic minority status, and housing type and transportation. A lower score indicates lower social vulnerability, whereas a higher score indicates higher social vulnerability, which might predispose a population to worse health outcomes. https://www.atsdr.cdc.gov/placeandhealth/svi

^§§^ https://www2.census.gov/geo/pdfs/maps-data/maps/reference/us_regdiv.pdf

^¶¶^ Underlying medical conditions were coded as not present if they were either specifically marked as absent or if they were not noted in the child’s medical record. The reference group for each comparison is defined by those who did not have the listed underlying health condition.

*** Immunocompromising conditions included active or previous oncologic disorder or nononcologic immunosuppressive disorder (including solid organ transplant, HIV or AIDS, primary immunodeficiency, bone marrow transplant for nononcologic disease, and other disorder requiring treatment that suppresses the immune system).

### Vaccine Effectiveness

VE of original monovalent mRNA COVID-19 vaccines against COVID-19–related hospitalization was 52% (95% CI = 33–66) when the most recent vaccine dose was received 7–119 days before hospitalization, 19% (95% CI = 2–32) when it was received 120–364 days before hospitalization, and 31% (95% CI = 18–43) if the last dose was received any time within the previous year. VE against critical COVID-19–related illness was 57% (95% CI = 21–76) when the last dose was 7–119 days before hospitalization, not significant when it was received 120–364 days before hospitalization, and 38% (95% CI = 15–55) when the most recent dose was received at any point within the previous year. During the peak of pediatric COVID-19 hospitalizations (December 19, 2021–March 19, 2022), VE was 55% (95% CI = 38–67) against COVID-19–related hospitalizations when the last dose was received a median of 129 days before hospitalization (IQR = 47–198 days) and 79% (95% CI = 59–89) against critical COVID-19–related illness when the last dose was received a median of 132 days before hospitalization (IQR = 46–215) (Supplementary Table, https://stacks.cdc.gov/view/cdc/152988). Estimates were similar after excluding children and adolescents with documented immunocompromising conditions (Table 3).

**TABLE 3:** Durability of effectiveness of original monovalent mRNA COVID-19 vaccination against hospitalization and critical illness for COVID-19 among pediatric patients aged 5–18 years, by age, vaccination timing, and patients without documented immunocompromising conditions — Overcoming COVID-19 Network, 34 pediatric hospitals, 26 states, December 19, 2021–October 29, 2023

Subgroup*	No. vaccinated/Total no. (%)		Interval from last vaccine dose to hospitalization, days, median (IQR)	VE against COVID-19 hospitalization, % (95% CI)^†^
	Case-patients	Control patients		
**Hospitalizations among all patients**				
Any original monovalent dose	371/1,508 (25)	529/1,739 (30)	169 (86 to 237)	31 (18 to 43)
7–119 days since last dose	94/1,231 (8)	207/1,417 (15)	53 (32 to 86)	52 (33 to 66)
120–364 days since last dose	277/1,414 (20)	322/1,532 (21)	212 (169 to 275)	19 (2 to 32)
Any dose, ages 5–11 yrs	114/832 (14)	207/1,013 (20)	120 (46 to 224)	40 (22 to 53)
Any dose, ages 12–18 yrs	257/676 (38)	322/726 (44)	181 (121 to 245)	28 (6 to 44)
**Hospitalizations among patients without documented immunocompromising conditions** ^§^ **^,^** ^¶^				
Any original monovalent dose	295/1,244 (24)	468/1,579 (30)	173 (88 to 243)	34 (22 to 44)
7–119 days since last dose	62/1,011 (6)	183/1,294 (14)	53 (33 to 84)	61 (40 to 75)
120–364 days since last dose	233/1,182 (20)	285/1,396 (20)	213 (171 to 278)	17 (0 to 31)
Ages 5–11 yrs	84/684 (12)	191/933 (20)	120 (46 to 222)	48 (29 to 61)
Ages 12–18 yrs	211/560 (38)	277/646 (43)	187 (129 to 252)	23 (2 to 40)**
**Critical illness**^††^ **among all patients**				
Any original monovalent dose	65/278 (23)	91/307 (30)	175 (79 to 253)	38 (15 to 55)
7–119 days since last dose	16/229 (7)	35/251 (14)	51 (36 to 74)	57 (21 to 76)^§§^
120–364 days since last dose	49/262 (19)	56/272 (21)	218 (172 to 287)	25 (–9 to 49)^§§^
**Critical illness**^††^ **among patients without documented immunocompromising conditions**^§^				
Any original monovalent dose	59/253 (23)	85/288 (30)	171 (73 to 247)	36 (17 to 50)
7–119 days since last dose	13/207 (6)	34/237 (14)	51 (36 to 71)	63 (35 to 79)**
120–364 days since last dose	46/240 (19)	51/254 (20)	218 (170 to 287)	16 (–20 to 41)**^,§§^

**Abbreviation**: VE = vaccine effectiveness.

* All analyses excluded patients who received a bivalent vaccine dose (43 case-patients and 58 control patients). Models examining VE by time since last dose incorporated a three-level categorical predictor variable (unvaccinated, last monovalent dose 7–119 days before hospitalization, and last original monovalent dose 120 –364 days before hospitalization) to obtain VE estimates for each interval range. Models examining VE by age were stratified by age group (5–11 years and 12–18 years). All children who had received any original monovalent dose received their last dose within the previous year before hospitalization (<365 days).

^†^ All models controlled for underlying medical condition, continuous age (in years), month and year of hospital admission, U.S. Census Bureau region, continuous social vulnerability index (range = 0–1), and race and ethnicity (categorized as non-Hispanic White, non-Hispanic Black or African-American, Hispanic or Latino, and other, multiple races, or unknown. Hospital site of enrollment was incorporated as a repeated effect.

^§^ This analysis excludes an additional 264 case-patients and 160 control patients who had documented immunocompromising conditions, yielding 1,244 case-patients and 1,579 control patients without any documented immunocompromising condition.

**^¶^** Immunocompromising conditions included active or previous oncologic disorder or immunosuppressive disorder (defined as solid organ transplant, HIV or AIDS, primary immunodeficiency, bone marrow transplant for nononcologic disease, or other disorder requiring treatment that suppresses the immune system).

** Where models did not converge, subvariant period (BA.1: December 19, 2021–March 19, 2022 and BA.2/BA.4/BA.5/XBB.1.5/XBB.1.6: March 20, 2022–October 29, 2023) was substituted as a covariate in place of month and year of hospital admission.

^††^ Critical illness was defined as illness resulting in noninvasive ventilation, invasive mechanical ventilation, receipt of vasoactive infusions, extracorporeal membrane oxygenation, or death. Both case-patients and control patients were required to have met this definition to be included in this subanalysis.

^§§^ Some estimates are imprecise (where 95% CIs were wider than 50%), which might be due to a relatively small number of persons in each level of vaccination or case status. This imprecision indicates that the actual VE could be substantially different from the point estimate shown, and estimates should therefore be interpreted with caution. Additional data accrual could allow more precise interpretation.

## Discussion

During the period of SARS-CoV-2 Omicron predominance, receipt of ≥2 original monovalent COVID-19 vaccine doses was associated with fewer COVID-19–related hospitalizations in children and adolescents aged 5–18 years; however, protection from original vaccines was not sustained over time, necessitating increased coverage with updated vaccines. Most children and adolescents in this analysis who were hospitalized with COVID-19 were unvaccinated, and few had received updated vaccine doses despite a high prevalence of underlying comorbidities associated with more severe disease. Vaccination frequency declined with increasing social vulnerability, highlighting disparities in vaccination coverage comparable with published estimates from at least one other U.S. public health surveillance network (*5*). This finding might be driven by factors including vaccine hesitancy or barriers to accessing vaccines among more vulnerable populations (*5*).

VE of original monovalent doses against COVID-19–related pediatric hospitalizations was lower than previous VE estimates reported by the Overcoming COVID-19 Network before Omicron emergence (*2*). However, VE estimates from this report among children and adolescents hospitalized during December 19, 2021–March 19, 2022, were similar to previously published VE estimates from this network among children and adolescents hospitalized within the same date range (*2*). In a separate U.S. study of children and adolescents aged 5–15 years, VE against symptomatic SARS-CoV-2 infections was reported to wane in the months after a second dose, with improved VE observed after receipt of a booster dose (*4*). Effectiveness of bivalent vaccine formulations against pediatric hospitalizations was not estimable in this investigation; however, two recent studies report that receipt of a bivalent vaccine was associated with higher VE against symptomatic pediatric infections (*6*) and COVID-19–related hospitalizations in immunocompetent adults (*7*).

### Limitations

The findings in this report are subject to at least four limitations. First, SARS-CoV-2 infection-induced immunity was not assessed (*8*); increased seroprevalence after Omicron BA.1 emergence (*9*) might have influenced observed VE. Second, limited viral sequencing data prevented consideration of subvariant-attributed immune evasion (*10*). Third, limited coverage with bivalent vaccines and currently recommended updated monovalent vaccines precluded the estimation of VE of these formulations. Finally, previously healthy children and adolescents accounted for <20% of case-patients, limiting generalizability.

### Implications for Public Health Practice

Among approximately 1,500 children and adolescents aged 5–18 years with a COVID-19–related hospitalization, including nearly 300 with critical illness, original monovalent COVID-19 vaccines were associated with fewer hospitalizations, particularly within the first 4 months after vaccination. To address low coverage of updated vaccines and waning effectiveness of the original monovalent vaccine, children and adolescents should remain up to date with COVID-19 vaccination, including the current CDC recommendation for all persons aged ≥6 months to receive vaccination with updated (2023–2024) COVID-19 vaccines (*1*).

## Footnotes

† A comprehensive listing of COVID-19 vaccination recommendations from the Advisory Committee on Immunization Practices is available. https://www.cdc.gov/vaccines/hcp/acip-recs/vacc-specific/covid-19.html

§ The original monovalent vaccine was administered for all COVID-19 vaccinations until the bivalent formulation was authorized (on September 1, 2022, for third or higher doses for those aged >12 years; October 12, 2022, for third or higher doses for children aged 5–11 years; and April 22, 2023, for first or second doses for all eligible ages).

¶ To use all available data, this investigation included children and adolescents admitted through October 29, 2023, which included September 11, 2023–October 29, 2023, when children and adolescents were eligible to receive updated monovalent vaccines specific for the Omicron XBB lineage. However, no child or adolescent in this investigation had received an updated monovalent dose before the October 29, 2023, cutoff date.

** Children and adolescents were enrolled from 34 hospitals in 26 states across all four U.S. Census Bureau regions. *Northeast*: Boston Children’s Hospital (Massachusetts), Children’s Hospital of Philadelphia (Pennsylvania), Cooperman Barnabas Medical Center (New Jersey), and Columbia University Irving Medical Center/New York-Presbyterian (New York); *Midwest*: Akron Children’s Hospital (Ohio), Children’s Hospital of Michigan (Michigan), Children’s Mercy Kansas City (Missouri), Children’s Nebraska (Nebraska), Cincinnati Children’s Hospital Center (Ohio), C.S. Mott Children’s Hospital (Michigan), Lurie Children’s Hospital (Illinois), Mayo Clinic (Minnesota), Minnesota Masonic (Minnesota), Nationwide Children’s Hospital (Ohio), and Riley Children’s (Indiana); *South*: Arkansas Children’s Hospital (Arkansas), Children's of Alabama (Alabama), Children’s Healthcare of Atlanta, Emory University (Georgia), Children’s Hospital of New Orleans (Louisiana), Children’s Medical Center of Dallas (Texas), Holtz Children’s Hospital (Florida), Medical University of South Carolina Children’s Health (South Carolina), Monroe Carell Jr. Children's Hospital at Vanderbilt (Tennessee), Texas Children’s Hospital (Texas),University of Mississippi Medical Center (Mississippi), and University of North Carolina at Chapel Hill Children’s Hospital (North Carolina); *West*: Children’s Hospital Colorado (Colorado), Children’s Hospital Los Angeles (California), Oregon Health & Science University Doernbecher Children’s Hospital (Oregon), Primary Children's Hospital (Utah), Seattle Children’s (Washington), University of California, San Francisco Benioff Children's Hospital Oakland (California), University of California San Diego-Rady Children’s Hospital (California), and University of California, San Francisco Benioff Children's Hospital (California).

†† Case-patients received a positive result for a SARS-CoV-2 nucleic acid amplification test (NAAT) or antigen test result 10 days before or within 72 hours after admission, with COVID-19 as the primary reason for hospitalization (directly or as an exacerbation of an underlying disease).

§§ Control patients matched to cases (1:1) by site, age group, and date of admission (within 3 weeks). COVID-19–like illness among control patients was defined as one or more of the following <14 days of hospitalization: fever, cough, shortness of breath, loss of taste or smell, new or elevated respiratory support, new pulmonary findings on chest imaging, and gastrointestinal symptoms. Control patients received negative test results for SARS-CoV-2 by NAAT during or ≤7 days before hospital admission, with no positive NAAT/antigen test result <3 days after hospitalization.

¶¶ Patients who had an incomplete COVID-19 mRNA vaccination series included those who received only 1 dose of an mRNA primary series or whose last dose was too recent (second dose was completed within 14 days of hospitalization or third or higher dose was received within 7 days of hospitalization). Those excluded because of unverifiable vaccination status include those whose vaccination status could not be verified through source documentation (such as state immunization information systems, electronic medical records, or pediatrician records) or plausible self-report, whereby a parent or caregiver provided the date and location of dose.

*** Immunocompromising conditions included active or previous oncologic disorder or nononcologic immunosuppressive disorder (including solid organ transplant, HIV or AIDS, primary immunodeficiency, bone marrow transplant for nononcologic disease, and other disorder requiring treatment that suppresses immune system).

††† Analyses included time since last dose as a multilevel categorical predictor and used the following cutoffs: 14–119 days for second dose or 7–119 days for a third or higher dose, and 120–364 days for all second or higher doses. The interval between receipt of the last dose and hospitalization was calculated as the number of inclusive days between those events. Models examining VE by age were stratified by age group (ages 5–11 years and 12–18 years).

§§§ Multivariable models controlled for the presence of at least one underlying medical condition, continuous age in years, month and year of hospital admission, U.S. Census Bureau region, continuous SVI ranging between 0 and 1, and race and ethnicity, categorized as non-Hispanic White, non-Hispanic Black or African-American, Hispanic or Latino, and other races, multiple races, or unknown.

¶¶¶ 45 C.F.R. part 46.102(l)(2), 21 C.F.R. part 56; 42 U.S.C. Sect. 241(d); 5 U.S.C. Sect. 552a; 44 U.S.C. Sect. 3501 et seq.

**** Initial inclusion criteria were met by 1,815 potential case-patients and 2,087 potential control patients. Among potential enrollees, 264 case-patients and 290 control patients were excluded, based on receipt of last vaccine dose ≥365 days before hospitalization (155 case-patients and 143 control patients), COVID-19 hospitalization within 60 days (13 case-patients and one control patient), incomplete vaccination or dose too recent (91 case-patients and 136 control patients), and unverifiable vaccination status through source documentation or plausible self-report (five case-patients and 10 control patients).

††††
https://www2.census.gov/geo/pdfs/maps-data/maps/reference/us_regdiv.pdf
